# Antitumor effect of cetuximab in combination with S-1 in EGFR-amplified gastric cancer cells

**DOI:** 10.3892/ijo.2011.1279

**Published:** 2011-12-01

**Authors:** KAZUMASA FUKUDA, YOSHIRO SAIKAWA, MASASHI TAKAHASHI, TSUNEHIRO TAKAHASHI, NORIHITO WADA, HIROHUMI KAWAKUBO, HIROYA TAKEUCHI, YUKO KITAGAWA

**Affiliations:** Department of Surgery, School of Medicine, Keio University, Tokyo 160-8582, Japan

**Keywords:** human epidermal growth factor receptor, cetuximab, S-1, gastric cancer, combination therapy

## Abstract

Overexpression of human epidermal growth factor receptor (EGFR) has been detected in gastric cancer (GC) and is associated with poor outcomes. Combination treatment regimens with EGFR-targeting agents and cytotoxic agents are considered to be a potential therapeutic option for EGFR-overexpressing GC. Herein, we have investigated the effects of combination treatment with the oral fluoropyrimidine S-1 and the EGFR-targeting agent cetuximab in GC cells with or without EGFR overexpression. EGFR expression was determined by FACS and quantitative PCR in GC cells. Experimental 5-fluorouracil (5FU) was used instead of S-1 for *in vitro* experiments. The efficacy of 5FU or cetuximab monotherapy or combination 5FU/cetuximab therapy was examined *in vitro* and *in vivo*. Clinical specimens were examined for EGFR by immunohistochemistry (IHC). EGFR expression score was defined as strong membrane and cytoplasmic staining in at least 50–75% of cells. The combination of 5FU and cetuximab synergistically inhibited cell proliferation and exhibited an enhanced proapoptotic effect in GC cells with EGFR overexpression. Cetuximab also induced down-regulation of phosphorylation of EGFR and AKT, leading to diminished signaling. The antitumor effect of the combination of S-1 and cetuximab *in vivo* was also greater than that of either drug alone. Our preclinical findings thus indicate that the combination of S-1 and EGFR-targeting therapy is a promising treatment option for GC with EGFR overexpression.

## Introduction

Adenocarcinoma of the stomach is the leading cause of gastrointestinal cancer in the world and is the second leading cause of cancer death worldwide (1). Currently available chemotherapy for advanced gastric cancer (GC) includes fluoropyrimidine-based agents. S-1 (TS-1) is an orally active combination of tegafur (a prodrug that is converted by cells to fluorouracil), gimeracil (an inhibitor of dihydropyrimidine dehydrogenase, which degrades fluorouracil) and oteracil (which inhibits the phosphorylation of fluorouracil in the gastrointestinal tract, thereby reducing the gastrointestinal toxic effects of fluorouracil) in a molar ratio of 1:0.4:1 (2,3). Results of the large-scale ACTS-GC (adjuvant chemotherapy trial of S-1 for gastric cancer) trial, which enrolled patients with locally advanced (stage II or III) GC who underwent D2 surgery, indicated that S-1 is an effective adjuvant treatment in this patient population. Other clinical trials have reported response rates of 30–50% for S-1 in advanced GC (4–6). Based on these results, S-1 is now recognized as one of the standard chemotherapeutic agents for this disease (7,8).

Although advanced GC is treated predominantly by combination chemotherapy regimens that include fluoropyrimidine derivatives, overall survival (OS) can still be improved (9,10). Investigational chemoradiotherapy regimens have also left room for improvement. In recent years, substantial advances have been made in the development of molecularly targeted therapies for various types of cancer. Targeted therapies block the growth of cancer cells by interfering with specific molecules required for carcinogenesis and tumor growth (11). Targeted cancer therapies have the potential to be more effective than current treatments and less harmful to normal cells.

Overexpression of human epidermal growth factor receptor 1 (EGFR; ErbB1; HER1 in humans) has been detected in approximately 30–70% of GC cases and is associated with poor outcomes and aggressive disease (12,13). EGFR status was reported to be significantly associated with OS and relapse-free survival (RFS) in both the surgery alone group and the S-1 group in the ACTS-GC study (Terashima M, *et al*, 2011 ASCO Meeting, 4013). Progress in the understanding of the involvement of the EGFR pathway in GC has recently been made. The binding of a ligand to the extracellular portion of EGFR results in phosphorylation of the tyrosine kinase domain located in the intracellular portion, resulting in activation of intracellular effectors involved in signaling, such as the G protein K-ras and the protein kinase RAF [Ras/mitogen-activated protein kinase pathway (MAPK)], as well as phosphoinositide 3-kinase (PI3K/Akt pathway). Cetuximab is a chimeric (mouse/human) IgG1monoclonal antibody directed against EGFR that is administered by intravenous infusion for treatment of metastatic colorectal cancer and head and neck cancer. Cetuximab has been proven to be effective in irinotecan-resistant metastatic colorectal cancer expressing EGFR, detected by immunohistochemistry (IHC), with response rates ranging from 8.8% when used as monotherapy to 22.9% when combined with irinotecan (14,15). Despite recent advances in the molecular understanding of GC, there is a noticeable lack of targeted therapies in clinical development for this malignancy.

In this study, we investigated whether cetuximab alone or in combination with S-1 can be used in the treatment of EGFR-overexpressing GC in cell culture and xenograft models as an indication of its potential efficacy for treating patients with GC.

## Materials and methods

### Cell culture and reagents

Human GC cell lines (MKN45, KATOIII, MKN74, MKN28 and MKN1) were obtained from the Japanese Cancer Research Resources Bank (Tokyo, Japan). MKN45 and KATOIII cells were derived from poorly-differentiated adenocarcinomas. MKN74 and MKN28 cells were derived from moderately- and well-differentiated adenocarcinomas, respectively. The MKN1 cell line is a gastric adenocarcinoma cell line obtained from a metastatic lymph node and has the ability to differentiate into either adenomatous or squamous cells. The KGC01 line was obtained from a patient with clinically diagnosed GC with a pathological diagnosis of type 4 carcinoma (pT4N1H0CY1M0/stage IV); this patient provided informed consent (approved by the ethics committee of Keio University; no. 17–47). These cell lines were cultured in RPMI-1640 (Sigma, St. Louis, MO) containing 10% fetal bovine serum (FBS; Invitrogen, Carlsbad, CA) and penicillin-streptomycin mixed solution (penicillin 10,000 units/ml, streptomycin 10,000 μg/ml; Nacalai Tesque, Inc., Kyoto, Japan). In all experiments, cells were cultured at 37˚C in a humidified 5% CO_2_/95% air atmosphere. Cetuximab (Erbitux, Merck, Lyon, France) was obtained from Bristol-Myers Squibb Co. (New York, NY), 5FU was obtained from Kyowa Hakko Kirin Co., Ltd. (Tokyo, Japan) and tegafur, gimeracil and oteracil, all of which are components of S-1, were synthesized by Taiho Pharmaceutical.

### Analysis of EGFR expression

To detect expression of EGFR, cells were removed from the culture dish using trypsin and EDTA, pelleted by centrifugation, washed with phosphate-buffered saline (PBS) and resuspended at 37˚C in Hanks' balanced salt solution (HBSS) containing 2% FBS and 10 mM HEPES (Invitrogen). Cells were incubated with Blocking One solution (Nacalai Tesque) for 20 min at room temperature. They were then washed and incubated with anti-human EGFR antibody conjugated with FITC (BD Pharmingen, San Jose, CA) for 30 min at 4˚C. Finally, cells were subsequently counterstained with 1 μg/ml propidium iodide (PI; Sigma) to label dead cells. Then, 1×10^6^ viable cells were analyzed using a FACSVantage™ SE (Becton-Dickinson, San Jose, CA). The distribution of cells was analyzed using FlowJo software (Tomy Digital Biology, Tokyo, Japan).

### Quantitative RT-PCR

otal RNA from cells was extracted using an RNeasy Mini Kit (Qiagen, Hilden, Germany) according to the manufacturer's protocol. The concentration of total RNA was determined using a NanoDrop ND-1000 (NanoDrop Technologies, San Diego, CA). Briefly, purified total RNA was reverse-transcribed to generate double-stranded cDNA using Eagle Taq Master Mix with ROX (Roche Applied Science, Indianapolis, IN) and the expression of human EGFR was analyzed using the Applied Biosystems 7500 Fast Real-Time PCR System (Applied Biosystems, Foster City, CA). TaqMan gene expression assay primers and probe mixes were used for GAPDH and EGFR (assay IDs Hs99999905_m1 and Hs01076078_m1, respectively; Applied Biosystems). GAPDH was detected using TaqMan primers and probes and was used as the control gene. The thermal cycling reaction included incubation at 95˚C for 20 sec and 40 cycles of 95˚C for 3 sec and 60˚C for 30 sec. Data were collected using analytical software (Applied Biosystems). Using the ΔΔC_T_ method, the expression level of each gene was determined relative to the value of the expression of the gene in TMK-1 cells.

### DNA extraction and K-ras mutation analysis

DNA was extracted from GC cell lines using a QIAmp DNA Mini kit (Qiagen, Duesseldorf, Germany). A NanoDrop ND-1000 (NanoDrop Technologies) was used to evaluate the concentration of the samples. The reaction mixes contain a single primer set specific for either the wild-type or mutated sequences in codons 12 and 13 of K-ras. Direct sequencing was done using a Big Dye Terminator cycle sequencing kit (Applied Biosystems) and analyzed on an ABI PRISM 310 DNA Analyzer automated sequencer (Applied Biosystems).

### Survival studies with anticancer agents

Cells were plated in 96-well micro-plates and cultured for 12 h before exposure to various concentrations of compounds for 72 h. Cells were quantified using the WST-8 assay. The optical density (OD) value was detected by Rainbo Sunrise (Wako Pure Chemical Industries, Ltd., Osaka, Japan). The rate of inhibition was calculated as follows: % inhibition = (OD of treated group − blank)/OD of control group × 100. The concentration of tested drugs resulting in 50% growth inhibition (IC_50_) was calculated. Data were analyzed to determine the combination index (CI), a well-established index of the interaction between two drugs (16). CI values of <1, 1 and >1 indicate synergistic, additive and antagonistic effects, respectively.

### Phosphorylation activity assay

To evaluate the dependency of cetuximab activity on EGFR and AKT phosphorylation, cells were exposed to 3.97 μM cetuximab for 72 h before they were collected. Cells were washed in PBS and fixed with 2% paraformaldehyde (PFA) in a 37˚C water bath for 10 min. Then, cells were washed with PBS and pelleted by centrifugation (800 × g) for 5 min and the supernatant was removed. Cells were mixed to disrupt the pellet and permeabilized by adding 500 μl of 90% methanol (for 1–10×10^6^ cells) and incubated on ice for 15 min. After blocking on ice for 10 min, cells were then washed and incubated with primary antibodies against EGFR, AKT, phospho-EGFR (Tyr1068) and phospho-AKT (Ser473) (Cell Signaling Technology, Inc., Danvers, MA) for 60 min at room temperature. Cells were washed with PBS before incubation for 30 min with Alexa Fluor 488 donkey anti-rabbit IgG antibody (Invitrogen). Then, each sample was analyzed using a FACSCalibur™ (Becton-Dickinson). Cell distribution was analyzed using FlowJo software (Tomy Digital Biology).

### Apoptosis assay

For apoptosis assays, the supernatant was aspirated and cells were resuspended in 150 μl binding buffer, and stained with 5 μl Annexin V-FITC and 5 μl PI at room temperature for 25 min in the dark. After incubation, cells were processed as directed in the kit instructions (Annexin V-FITC Apoptosis Detection Kit I, BD Pharmingen) and analyzed using a FITC signal detector and PI detector using a flow cytometer (FACSCalibur™) and Cell Quest software (Becton-Dickinson).

### In vivo multiple drug assay

MKN28 cells (1×10^6^) were implanted s.c. into the axilla of 6-week-old male athymic nude mice. Drug administration was initiated when tumors in each group achieved an average volume of 333±2.16 mm^3^. Mice were randomly allocated to control and treatment groups. Treatment groups consisted of control, S-1 alone, cetuximab alone and combination S-1/cetuximab. Each treatment group included 8 mice. S-1 was administered at a dose of 6.9 g/kg/day and given by oral gavage daily for 14 days. Cetuximab (40 mg/kg/day) was given i.p. on Days 1, 4, 8 and 14. Control animals received sterile PBS administration. Tumor volume was determined from caliper measurements of tumor length (L) and width (W) according to the formula LW^2^/2 and on body weights acquired every 3 days and on the day of evaluation. Both tumor size and body weight were measured three times per week. The percentage of tumor growth inhibition (TGI%) was calculated as follows: TGI (%) = [1 − (tumor volume of treatment group on evaluation day − tumor volume of treatment group on Day 1)/(tumor volume of control group at evaluation day − tumor volume of control group on Day 1)] × 100. The percentage of body weight change (BWC%) was calculated as follows: BWC (%) = [(BW on evaluation day) − (BW on Day 1)]/(BW on Day 1) × 100.

### Immunohistochemical analysis

Samples were fixed with 4% PFA for 24 h at RT. Immunohistochemical staining for EGFR was performed on 4-μm thick sections placed on pre-coated slides with APS (Matsunami Glass Ind., Ltd., Osaka, Japan). Briefy, slides were incubated with blocking reagent-N101 (Wako Pure Chemical Industries, Tokyo, Japan) for 20 min. After rinsing in PBST, avidin and biotin blocking was performed for 15 min each. Slides were incubated with anti-human EGFR monoclonal antibody (Cell Signaling Technology, Inc.). EnVision™+, Rabbit/HRP (Dako, Glostrup, Denmark) was then used as secondary antibody for 30 min. DAB staining reactions were conducted for 10 min. Slides were counterstained with haematoxylin. Finally, slides were cover-slipped with aqueous mounting medium (Aquatex^®^, Merck). Specimens were analyzed under a light microscope, and EGFR positivity was defined as strong membrane and cytoplasmic staining in at least 50–75% of cells.

### Statistical methods

All data were expressed as the mean ± SD. Statistically significant differences were determined using two-tailed Student's t-test or χ^2^ test. P-values <0.05 were considered statistically significant.

## Results

### Profile of EGFR amplification in GC cells

**Figure 1:**
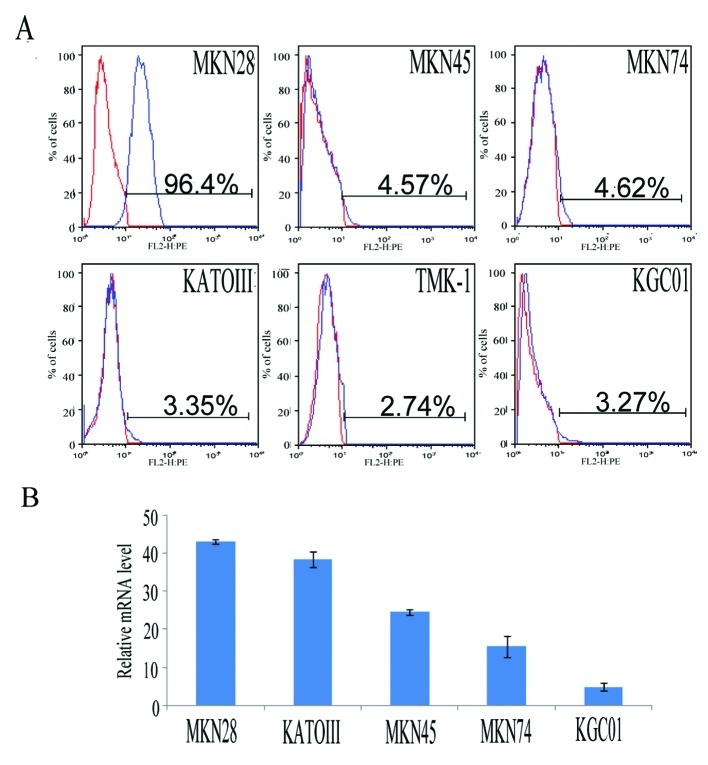
Analysis of EGFR expression in human GC cell lines. (A) Flow cytometric analysis of EGFR expression in human GC cell lines. Cells were treated with monoclonal antibodies against human EGFR. Expression levels were estimated by the intensity of fluorescence with phycoerythrin (PE) in the samples (red histogram, isotype control; blue histogram, cetuximab). (B) Relative mRNA expression of EGFR in GC cell lines was determined using a quantitative real-time RT-PCR amplification analysis. Results were analyzed using the relative quantification (ΔΔC_T_) method.

To evaluate EGFR amplification and K-ras mutation status, six GC cell lines (MKN28, MKN45, MKN74, KATOIII, TMK-1 and KGC01) were examined for their expression of EGFR protein and mRNA by flow cytometry and real-time PCR and for mutation analysis by direct sequencing. FACS analysis revealed that EGFR expression was significantly higher in MKN28 cells compared to other cell lines (P<0.05) (Fig. 1A). The EGFR mRNA expression ratio of each cell line was determined relative to the value of TMK-1. The EGFR mRNA level was 43.08±0.53-fold for MKN28 cells, 24.57±0.62-fold for MKN45 cells, 15.52±2.79-fold for MKN74 cells, 38.19±2.07-fold for KATOIII cells and 4.90±1.12-fold for KGC01 cells (Fig. 1B). K-ras mutation status for each cell line is shown in Table I. Among the GC cell lines examined, EGFR protein and mRNA were overexpressed in MKN28 cells, while KATOIII cells showed amplification of EGFR mRNA, but were negative for EGFR protein expression.

### Synergistic anti-proliferative effects of 5FU and cetuximab in EGFR-amplified GC cells

**Figure 2:**
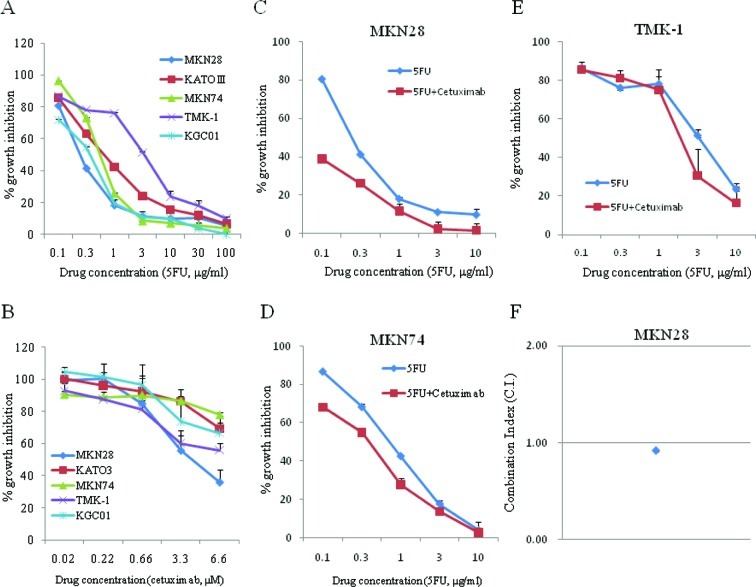
Anti-proliferative effects of 5FU monotherapy, cetuximab monotherapy and combination 5FU/cetuximab *in vitro*. (A, B) GC cells were maintained in supplemented medium for 12 h and then incubated with 5FU (0.1–100 μg/ml) or cetuximab (0.02–6.6 μM) for 72 h. (C–E) EGFR-amplified MKN28 cells or non-EGFR-amplified MKN74 and TNK-1 cells were incubated for 72 h with 5FU (0–10 μg/ml) and cetuximab at a fixed cetuximab concentration of 3.97 μM, after which cell viability was measured. (F) The interaction between the two agents was evaluated on the basis of the CI. CI values of <1, 1 and >1 indicate synergistic, additive and antagonistic effects, respectively. Data are means of triplicates from a representative experiment.

The effects of 5FU or cetuximab monotherapy or combination 5FU/cetuximab therapy on the growth of GC cells with and without EGFR amplification were evaluated. 5FU was used instead of S-1 for these *in vitro* experiments, since tegafur, a component of S-1, is metabolized to 5FU in the liver. The combined effect of 5FU and cetuximab was evaluated on the basis of the CI. 5FU monotherapy inhibited the proliferation of GC cells, although the IC_50_ values varied significantly between the individual cell lines (Fig. 2A and B). On the other hand, EGFR-amplified MKN28 cells showed only sensitive to cetuximab in a concentration-dependent manner compared with other GC cells (Fig. 2C). The combination of 5FU and cetuximab exhibited a synergistic inhibitory effect on the growth of EGFR-amplified MKN28 cells (C.I. value = 0.92±0.015), but not on cells without EGFR amplification, including MKN74 and TMK-1 cells (Fig. 2C–F).

### Effect of cetuximab on EGFR and AKT signaling in GC cells

**Figure 3:**
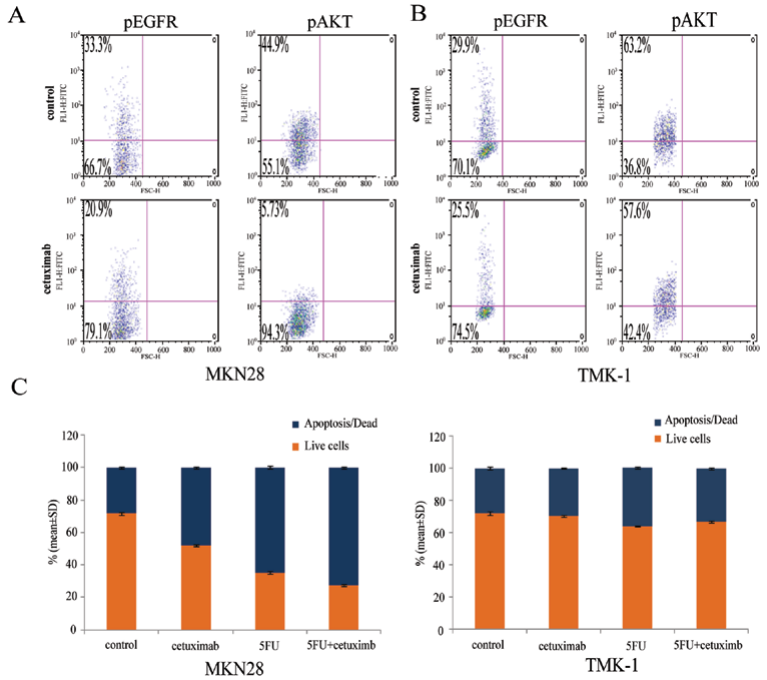
Effect on cell signaling and apoptosis. (A, B) Cells were treated with 3.97 μM cetuximab for 72 h. Decreased pEGFR and pAKT activity is observed following cetuximab treatment in EGFR-amplified MKN28 cells, but not in non-EGFR-amplified TMK-1 cells. (C) The effect of 5FU and cetuximab on apoptosis in EGFR-amplifed GC cells. MKN28 and TMK-1 cells were treated for 72 h with each agent alone or combination 5FU/cetuximab. The proportion of apoptotic cells was assessed by staining with FITC-conjugated Annexin V and PI followed by flow cytometry. Data are the means ± SD from three independent experiments.

EGFR can signal through the AKT or MAPK pathways (17). To explore the anti-proliferation mechanism of EGFR-targeted agents, we examined the effects of cetuximab on the EGFR/AKT signaling pathway. MKN28 and TMK-1 cells were treated with cetuximab for 72 h. In the EGFR-amplified cell line MKN28, cetuximab decreased both EGFR and AKT phosphorylation when compared with the isotype controls. In contrast, phosphorylation of EGFR or AKT was not affected by cetuximab in TMK-1 cells, in which EGFR is not amplified (Fig. 3A). These data indicate that cetuximab can suppress the activation of key pathways that are downstream of EGFR.

### Enhanced induction of apoptosis by combined 5FU and cetuximab in EGFR-amplified GC cells

To investigate the mechanism underlying the synergistic growth inhibition induced by combination of 5FU and cetuximab, we examined the effects of each agent alone and in combination on apoptosis in GC cells. An assay based on the binding of Annexin V to the cell surface revealed that the frequency of apoptosis was markedly greater in EGFR-amplified cells treated with both 5FU and cetuximab than in cells treated with either agent alone (Fig. 3B). No such effect was observed in cells negative for EGFR amplification. These data indicate that the combination of 5FU and cetuximab exhibits an enhanced apoptotic effect in EGFR-amplified GC cells, but not in those without EGFR amplification.

### Effects of combination cetuximab and S-1 therapy on EGFR-overexpressing human GC xenograft models

**Figure 4:**
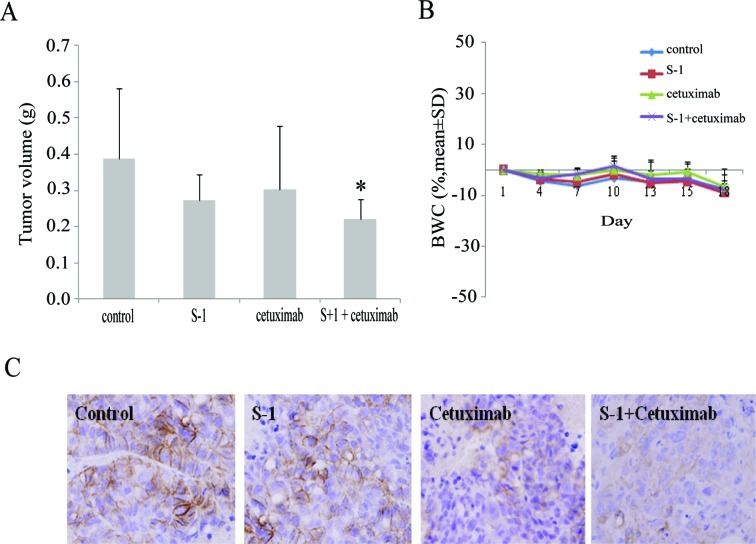
Antitumor activity of cetuximab and S-1 on tumor growth in an EGFR-amplified xenograft model. MKN28 cells (1×10^6^ cells with 50% Matrigel) were injected s.c. into nude mice and mice were randomized into four groups (n=8/group). Treatment started when tumors in each group achieved an average volume (333±2.16 mm^3^) with S-1 (6.9 mg/kg daily for 14 days), cetuximab (40 mg/kg i.p. on Days 1, 4, 8 and 14), sterile PBS (control) or combination S-1/cetuximab (same doses as above). Tumor sizes and body weights were measured every 3 days. (A) Student's t-test was used to compare tumor volume (g) between groups at the end of the experiment; results are presented as means. Error bars represent SD of the mean (^*^P<0.05). (B) All treatments were well tolerated by the mice, with no signs of toxicity or weight loss during therapy. (C) Tumor tissues of each treatment group were examined for expression of EGFR protein by IHC. ^*^Statistically significant.

The antitumor activities of cetuximab combined with chemotherapy were examined in an EGFR-overexpressing human GC xenograft model. Mice with tumors derived from MKN28 cells were divided into groups for treatment with vehicle, S-1, cetuximab, or combined S-1/cetuximab for 14 days. Tumor volume (TV) was evaluated between groups at the end of the experiment. The TV (g) for combined S-1/cetuximab was 0.22±0.05 g, whereas for control, S-1 and cetuximab alone was 20.0±1.96 g, 0.27±0.07 g and 0.30±0.17 g, respectively. Additionally, the TGI % for cetuximab combined with S-1 was 43.2%, while that for S-1 and cetuximab alone was 29.8 and 22.4%, respectively. Combination S-1/cetuximab therapy inhibited the growth of tumors formed by EGFR-amplified MKN28 cells compared to treatment with either agent alone (P<0.05) (Fig. 4A). All treatments were well tolerated by the mice, with no signs of toxicity or weight loss during therapy (Fig. 4B). Furthermore, tumors in each treatment group were examined for expression of EGFR protein by IHC. EGFR expression was decreased in the cetuximab alone and the S-1/cetuximab groups compared to the control and S-1 alone groups (Fig. 4C). Thus, the combination S-1/cetuximab therapy appears to result in an enhanced antitumor effect in EGFR-amplified GC xenografts, consistent with the results obtained *in vitro*.

### EGFR expression in patients with GC

**Figure 5:**
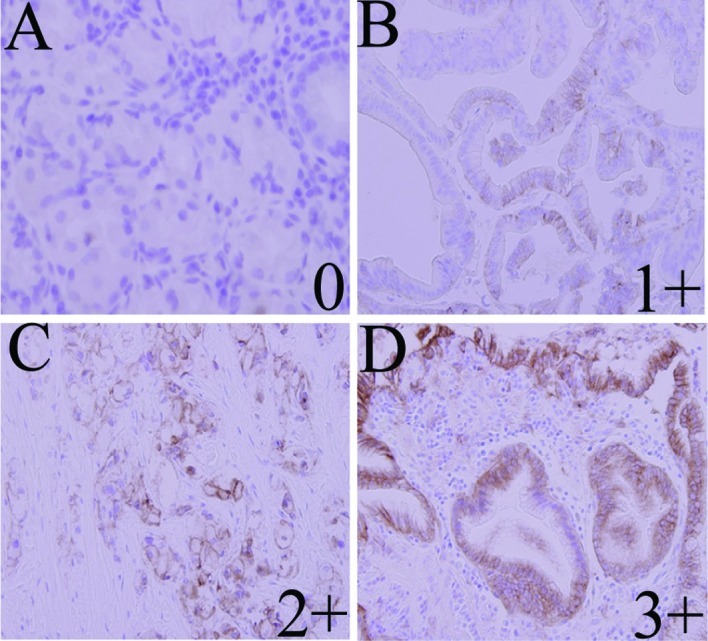
Image analysis of GC clinical specimens immunostained with anti-human EGFR monoclonal antibody. Immunohistochemical staining was performed by the peroxidase method. A–D were scored 0, 1+, 2+ and 3+, respectively, in quantitative analysis, according to the EGFR pharmDx™ Interpretation Manual (magnification, ×200).

Among the 40 specimens, the median age of patients was 67 years (range, 32–94 years); 20 patients had differentiated carcinoma and the other half had undifferentiated carcinoma. Formalin-fixed paraffin-embedded specimens from all 40 patients were examined for EGFR by IHC (Fig. 5). Analysis of EGFR protein expression by microscopic observation revealed a score of 0 in24 cases (60%), 1+ in 5 cases (12.5%), 2+ in 6 cases (15%) and 3+ in 5 cases (12.5%). EGFR reactivity was not correlated with gender, age, differentiation, lymph node metastasis or distant metastasis. Significantly more clinicopathological stage III/IV cases had EGFR-positive disease (30%) compared to stage I/II cases (10%, P=0.0088) (Table II). These results indicate that EGFR positivity is associated with advanced disease in GC.

## Discussion

EGFR amplification has been suggested to be associated with prognosis in gastrointestinal carcinoma. Cetuximab is a chimeric anti-EGFR monoclonal antibody that inhibits signaling pathways affecting cellular growth, differentiation and proliferation (18). Cetuximab is widely used as a standard therapy for EGFR-positive colorectal and head and neck cancer, and shows clinical efficacy both alone and in combination with chemotherapeutic agents (19–23). However, only limited evaluation of EGFR-targeted agents has been conducted in GC models and most such studies have been restricted to EGFR-amplified cells. Furthermore, the mechanisms of action of EGFR-targeted agents in combination with cytotoxic agents have remained unclear. In the present study, we have shown that the combination of S-1 (or 5FU) and EGFR-targeted therapy results in a synergistic antitumor effect in EGFR-amplified GC cells, but not in those lacking EGFR amplification.

To explore the efficacy of cetuximab alone or in combination with S-1 in EGFR-overexpressed GC, we evaluated the effect of cetuximab in a panel of molecularly characterized human GC cell lines. The level of EGFR protein expression was determined for a panel of six human GC cancer cell lines, and mRNA status was assessed by real-time PCR. Dose-response curves were generated to determine sensitivity to 5FU or cetuximab. Cetuximab had concentration-dependent anti-proliferative activity across the panel, with the greatest effects in EGFR-amplified MKN28 cells. In contrast, the EGFR low-expressing cell lines MKN74 and TMK-1 were less sensitive to cetuximab. Moreover, Fig. 2 shows that the combination of 5FU and cetuximab was highly synergistic in inhibiting cell growth, with a CI of <1 in MKN28. Cetuximab inhibited phosphorylation of EGFR (pEGFR) and AKT (pAKR) in EGFR-amplified MKN28 cells, but not in cells without EGFR amplification. Down-regulation of pEGFR and pAKR by cetuximab treatment may therefore enhance 5FU-induced apoptosis. Previous studies reported that activation of EGFR leads to downstream signaling that activates mitogenic and survival pathways, such as the MAPK and PI3-K/AKT pathways (24). Inhibition of these pathways by EGFR antagonists, such as cetuximab, can lead to induction of apoptosis and anti-proliferative effects (25). These results suggest that combination therapy may block the signaling pathways downstream of EGFR. Moreover, the efficacy of cetuximab monotherapy, S-1 monotherapy or combination S-1/cetuximab was examined in an EGFR-amplified xenograft model. In the MKN28 xenograft, combination S-1/cetuximab induced a near complete tumor regression in all treated mice. In the cetuximab monotherapy and combination S-1/cetuximab treatment groups, EGFR expression was down-regulated compared to that of the control and S-1 monotherapy groups, suggested that cetuximab may be blocking combination of ligand and EGFR-driven signaling. The combination therapy effects were more pronounced than either cetuximab or S-1 alone. EGFR expression was required to obtain a positive response to combination S-1/cetuximab, which is consistent with the results of numerous studies demonstrating that the antitumor activity of several anticancer agents increases when combined with cetuximab (26–34).

Clinical specimens from 40 patients with GC were examined for EGFR by IHC. EGFR expression was detected in 40% (16/40) of clinical specimens. Overall, 30% of clinicopathological stage III/IV patients demonstrated EGFR positivity (P=0.0088). These data indicate that EGFR expression is associated with advanced disease in GC. EGFR appears to be a potential target molecule for the treatment of GC. A recently reported phase II clinical trial showed a significant gain in OS for EGFR-positive patients with advanced GC who received combined treatment with cetuximab and FOLFOX6 (35). The present observations provide a rationale for clinical evaluation of combination chemotherapy with S-1 and EGFR-targeted agents according to EGFR amplification status.

In conclusion, the present results demonstrate that the combination cetuximab/S-1 can enhance S-1 antitumor activity. EGFR amplification is the best predictive marker for the anti-proliferative effects of combination chemotherapy with S-1 and cetuximab. The combination of cetuximab and S-1 may be a promising therapeutic strategy for some patients with EGFR-amplified GC.

## Figures and Tables

**Table I:** EGFR status and K-ras mutation in gastric cancer.

Cell line	EGFR protein expression (%, mean ± SD)	EGFR mRNA amplification (fold, mean ± SD)	K-ras mutation
MKN28	95.90±1.23	43.08±0.53	WT
MKN45	4.16±0.51	24.57±0.62	WT
MKN74	4.35±0.26	15.52±2.79	WT
TMK-1	2.43±0.32	1 (control)	WT
KATOIII	3.23±0.12	38.19±2.07	WT
KGC01	3.09±0.19	4.90±1.12	WT

EGFR protein expression on the cell surface was detected by flow cytometry. mRNA was purified from GC cell lines and EGFR amplification was analyzed using the Applied Biosystems 7500 Fast Real-Time PCR System. Mutation status of K-ras in cell lines was measured by ABI PRISM 310 DNA analyzer.

**Table II:** Association between EGFR status and clinicopathological characteristics of patients including gender, age, stage, differentiation and metastasis.

	Positive patients (%)	Negative patients (%)	P-value
Total	16 (40.0)	24 (60.0)	
Gender			
Male	14 (35.0)	14 (35.0)	0.078
Female	2 (5.0)	10 (25.0)	
Age			
<70	9 (22.5)	7 (17.5)	0.109
≥70	7 (17.5)	17 (42.5)	
Stage			
IA/IB/II	4 (10.0)	17 (42.5)	0.008
IIIA/IIIB/IIIC/IV	12 (30.0)	7 (17.5)	
Differentiation			
Differentiated	9 (22.5)	11 (27.5)	0.747
Undifferentiated	7 (17.5)	13 (32.5)	
Lymph node metastasis			
Absent	3 (7.5)	12 (30.0)	0.100
Present	12 (30.0)	13 (32.5)	
Distant metastasis			
Absent	1 (2.5)	2 (5.0)	0.999
Present	15 (37.5)	22 (55.0)	

## Abbreviations

GC: gastric cancer
EGFR: epidermal growth factor receptor
